# Combination of Anti-CD40 and Anti-CD40L Antibodies as Co-Stimulation Blockade in Preclinical Cardiac Xenotransplantation

**DOI:** 10.3390/biomedicines12081927

**Published:** 2024-08-22

**Authors:** Martin Bender, Jan-Michael Abicht, Bruno Reichart, Elisabeth Neumann, Julia Radan, Maren Mokelke, Ines Buttgereit, Maria Leuschen, Felicia Wall, Sebastian Michel, Reinhard Ellgass, Stig Steen, Audrius Paskevicius, Andreas Lange, Barbara Kessler, Elisabeth Kemter, Nikolai Klymiuk, Joachim Denner, Antonia W. Godehardt, Ralf R. Tönjes, Jonathan M. Burgmann, Constança Figueiredo, Anastasia Milusev, Valentina Zollet, Neda Salimi-Afjani, Alain Despont, Robert Rieben, Stephan Ledderose, Christoph Walz, Christian Hagl, David Ayares, Eckhard Wolf, Michael Schmoeckel, Paolo Brenner, Uli Binder, Michaela Gebauer, Arne Skerra, Matthias Längin

**Affiliations:** 1 Department of Anaesthesiology, University Hospital, LMU Munich, 81377 Munich, Germany; 2 Transregional Collaborative Research Center 127, Walter Brendel Centre of Experimental Medicine, LMU Munich, 81377 Munich, Germany; 3 Department of Cardiac Surgery, University Hospital, LMU Munich, 81377 Munich, Germany; 4 Munich Heart Alliance, German Center for Cardiovascular Research (DZHK), 81377 Munich, Germany; 5 Department of Cardiothoracic Surgery, Lund University and Skåne University Hospital, 22242 Lund, Sweden; 6 Institute of Molecular Animal Breeding and Biotechnology, Gene Center, and Department of Veterinary Sciences, LMU Munich, 81377 Munich, Germany; 7 Institute of Virology, Free University Berlin, 14163 Berlin, Germany; 8 Division of Haematology, Cell and Gene Therapy, Paul-Ehrlich-Institut, 63225 Langen, Germany; 9 Institute of Transfusion Medicine and Transplant Engineering, Hannover Medical School, 30625 Hannover, Germany; 10 Department for BioMedical Research (DBMR), University of Bern, 3008 Bern, Switzerland; 11 Graduate School for Cellular and Biomedical Sciences (GCB), University of Bern, 3008 Bern, Switzerland; 12 Institute of Pathology, Faculty of Medicine, LMU Munich, 81377 Munich, Germany; 13 Revivicor, Blacksburg, VA 24060, USA; 14 Center for Innovative Medical Models (CiMM), LMU Munich, 81377 Munich, Germany; 15 Interfaculty Center for Endocrine and Cardiovascular Disease Network Modelling and Clinical Transfer (ICONLMU), LMU Munich, 81377 Munich, Germany; 16 XL-protein GmbH, 85354 Freising, Germany; 17 Chair of Biological Chemistry, School of Life Sciences, Technical University of Munich, 85354 Freising, Germany

**Keywords:** CD40/CD40L, co-stimulation blockade, heart, orthotopic heart transplantation, xenotransplantation

## Abstract

The blockade of the CD40/CD40L immune checkpoint is considered essential for cardiac xenotransplantation. However, it is still unclear which single antibody directed against CD40 or CD40L (CD154), or which combination of antibodies, is better at preventing organ rejection. For example, the high doses of antibody administered in previous experiments might not be feasible for the treatment of humans, while thrombotic side effects were described for first-generation anti-CD40L antibodies. To address these issues, we conducted six orthotopic pig-to-baboon cardiac xenotransplantation experiments, combining a chimeric anti-CD40 antibody with an investigational long-acting PASylated anti-CD40L Fab fragment. The combination therapy effectively resulted in animal survival with a rate comparable to a previous study that utilized anti-CD40 monotherapy. Importantly, no incidence of thromboembolic events associated with the administration of the anti-CD40L PAS-Fab was observed. Two experiments failed early because of technical reasons, two were terminated deliberately after 90 days with the baboons in excellent condition and two were extended to 120 and 170 days, respectively. Unexpectedly, and despite the absence of any clinical signs, histopathology revealed fungal infections in all four recipients. This study provides, for the first time, insights into a combination therapy with anti-CD40/anti-CD40L antibodies to block this immune checkpoint.

## 1. Introduction

Cardiac xenotransplantation has seen notable improvement in the last few years and is becoming the most promising alternative to human heart allotransplantation [1,2]. In life-supporting pig-to-baboon experiments, consistent survival for up to nine months was achieved [3,4,5]. A cornerstone for cardiac xenotransplantation is the effective co-stimulation blockade of the CD40/CD40 ligand (CD40L *alias* CD154) pathway [1,6], which plays a pivotal role in both T cell-driven inflammation and humoral immune responses [7]. In 2022, for the first time, a 10-fold genetically modified porcine heart was transplanted into a human as an individual medical treatment after receiving approval by the United States Food and Drug Administration (FDA) and ethics committee [8]. The patient survived for two months with an immunosuppressive therapy based on the blockade of the CD40/CD40L axis [8,9]. As testing of the pig organ donor revealed later, porcine cytomegalovirus/porcine roseolovirus (PCMV/PRV) was transmitted to this patient and could have contributed to his early death [8].

As members of the tumor necrosis factor (TNF) receptor superfamily, CD40L and its receptor CD40 represent a key “immune checkpoint” [7]. However, the expression of CD40 and its counterpart CD40L differ depending on the type of immune cell [10,11,12,13]. CD40 is constitutively expressed on antigen-presenting cells such as B cells, dendritic cells, macrophages, monocytes, platelets, fibroblasts and epithelial as well as endothelial cells [10,11,12]. CD40L, on the other hand, is found on activated B and, in particular, T cells as well as platelets, and its expression can also be induced on monocytic cells, natural killer cells, mast cells and basophils [10,13].

Since many biological processes like humoral and cellular immunity, tissue inflammation, thrombosis, hematopoiesis and tumor cell fate are regulated by CD40/CD40L interaction [7], both cell surface receptors are under intensive investigation with regard to therapeutic intervention [7,10]. Indeed, the blockade of the CD40/CD40L pathway with either anti-CD40 or anti-CD40L monoclonal antibodies (Mabs) has demonstrated excellent therapeutic potential in preclinical non-human primate xenotransplantation models up to now [3,4,5,14,15,16,17]. However, despite such successful studies, it is still not known which Mab type in which dosage will be clinically preferable, and none of these Mabs has received clinical approval yet [10].

In this context, the initially promising clinical development of an anti-CD40L-specific therapy [18] has been challenged by the finding that the corresponding Mab hu5C8 (ruplizumab) was associated with an increased risk of thromboembolic events [16]. These side effects were later shown to be caused by the Fc region of the antibody, which activates platelets via an Fc receptor-dependent mechanism [19,20]. Interestingly, in some experimental studies the blockade of CD40L after both allo- and xenotransplantation was demonstrated to be more effective in prolonging graft survival compared to CD40 blockade [21,22,23]. Moreover, mounting evidence over the past years raised the possibility that the biology behind CD40/CD40L is more complicated than anticipated, such that blockade of CD40 versus blockade of CD40L is not mechanistically equivalent [24,25]. While CD40L—which also exists in a soluble form (sCD40L)—was believed for a long time to interact exclusively with CD40, and all the biological functions were attributed to this interaction, newer findings revealed the existence and functional role of alternative receptors, with some of them also involved in controlling the immune response [22,25,26,27,28].

Furthermore, apart from their mutual interaction as part of the co-stimulatory signaling cascade [29] both proteins, in particular CD40L, exert distinct autocrine functions on their respective host cells [30]. In fact, the receptor CD40 itself shows homotypic association, and cognate Mabs may have agonistic, antagonistic as well as CD40L-blocking activity [31,32], depending on their epitopes recognized. Therefore, there should be a difference between targeting either CD40 or CD40L with a potent Mab—in addition to the effects of epitope specificity—in order to achieve an efficient co-stimulation blockade for xenotransplantation. Beyond that, a combination of both types of antibodies may even have a synergistic effect.

To elucidate this notion, we used a combination therapy of a well-studied mouse/rhesus chimeric anti-CD40 IgG4 monoclonal antibody (anti-CD40 Mab) [11,31] with a humanized PASylated anti-CD40L antigen-binding fragment (anti-CD40L PAS-Fab) [3,33] derived from ruplizumab—which lacks Fc immune effector functions and antigen dimerization activity but has a strongly extended plasma half-life—as part of the immunosuppressive regimen for orthotopic pig-to-baboon cardiac xenotransplantation experiments. With this combination therapy, we aimed at potential synergistic effects of a combined CD40/CD40L co-stimulation blockade, while at the same time, avoiding the disadvantages of high-dose anti-CD40 or anti-CD40L monotherapies.

## 2. Materials and Methods

### 2.1. Animals

Hearts from six genetically modified piglets were transplanted into male captive-bred baboons. The piglets (German Landrace/Large White; blood group 0) were homozygous for α1,3-galactosyltransferase knockout (*GGTA1*-*KO*) and hemizygous transgenic for human CD46 (*hCD46*) and human thrombomodulin (*hTBM*) (Revivicor, Blacksburg, VA, USA and Institute of Molecular Animal Breeding and Biotechnology, Gene Center, LMU Munich, Munich, Germany). Six baboons (*Papio anubis* and *Papio hamadryas*; blood group A (*n* = 4) or AB (*n* = 2); German Primate Centre DPZ, Göttingen, Germany) served as recipients. The expression of *hCD46* and *hTBM* was verified post-mortem by immunohistochemistry.

The study was approved by the Government of Upper Bavaria (Regierung von Oberbayern (ROB); Munich, Germany). All animals were cared for and treated in accordance with the Guide for the Care and Use of Laboratory Animals (German Legislation for the Welfare of Laboratory Animals and US National Institutes of Health).

### 2.2. Virological Screening

Both the donor pigs and the baboon recipients were screened for PCMV/PRV using real-time PCR, nested-PCR and immunological methods like Western blot and peptide ELISA. The procedures were performed as described in parallel at the Free University Berlin [34] and the Paul-Ehrlich-Institute [35]. In addition, the donor pigs were tested for porcine circovirus 3 (PCV3), which was once transmitted to baboon recipients of pig hearts [36], as well as porcine lymphotropic herpesvirus-3 (PLHV-3) using PCR methods as described elsewhere [34].

### 2.3. Anesthesia, Surgical Procedures and Heart Preservation

After sedation, induction of anesthesia and endotracheal intubation of the animals [37], surgery was conducted as published in detail elsewhere [3].

In brief, after median sternotomy of the donor animal, the aorta was cross-clamped and antegrade non-ischemic preservation commenced immediately; continuous perfusion with 8 °C oxygenated, hyperoncotic solution containing albumin, hormones, nutrients and erythrocytes [38,39] was provided by an extracorporeal heart preservation system (University of Lund, Lund, Sweden) consisting of a pressure- and flow-controlled roller pump, an O_2_/CO_2_ exchanger, a leukocyte filter and a cooler/heater unit. During storage, the heart was preserved the same way and the perfusion pressure kept at 20 mmHg.

After median sternotomy in the baboon recipient, extracorporeal circulation was installed and started. Explantation of the recipient´s native heart and xenotransplantation followed the techniques of Lower and Shumway [40]. The donor heart was intermittently perfused for 2 min every 15 min during implantation.

### 2.4. Immunosuppression

For induction therapy, all animals received the B cell-depleting anti-CD20 Mab rituximab (Mabthera; Roche Pharma, Basel, Switzerland), ATG (thymoglobulin; Sanofi, Paris, France) and a combination of an anti-CD40 Mab (50 mg/kg body weight (bw); mouse/rhesus chimeric IgG4 clone 2C10R4, NIH Non-human Primate Reagent Resource; Mass Biologicals, Boston, MA, USA; courtesy of K. Reimann) and the anti-CD40L PAS-Fab (20 mg/kg bw; XL-protein, Freising, Germany).

**Figure 1:**
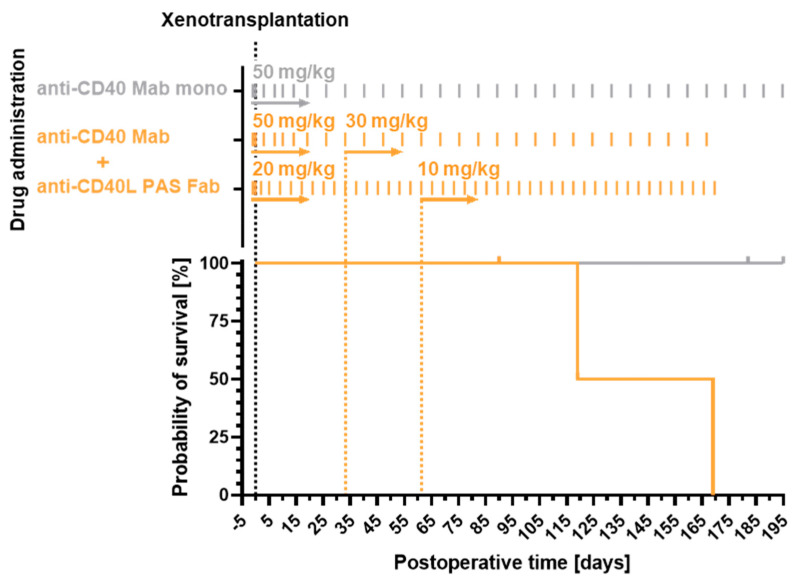
Dosing scheme (top) and survival (bottom) of the study group comprising six animals, which received anti-CD40 IgG4/anti-CD40L PAS-Fab combination therapy (orange). The dosage of the anti-CD40 Mab was reduced within 30 days from initially 50 to 30 mg/kg body weight (bw), and the dosage of the anti-CD40L PAS-Fab was lowered within 60 days from 20 to 10 mg/kg bw. In comparison with a previously reported [4] immunosuppressive regimen with the anti-CD40 Mab alone (gray), there was no significant difference in survival (Log-rank (Mantel–Cox) test, *n* = 10, *p* = 0.0896). In the current study group, two technical failures were excluded from the survival analysis. In the previously described group [4], two animals which were positive for PCMV/PRV were excluded. PCMV/PRV, porcine cytomegalovirus/porcine roseolovirus.

Immunosuppression was maintained with mycophenolate mofetil (CellCept; Roche Pharma), methylprednisolone (urbasone soluble; Sanofi) and the combination therapy from above comprising anti-CD40 Mab (50 mg/kg bw once weekly, reduced to 30 mg/kg bw after 1 month) and anti-CD40L PAS-Fab (20 mg/kg bw every 4 days, reduced to 10 mg/kg bw after 2 months) at successively decreased doses (Figure 1).

In addition, all animals received a therapeutic regimen to slow down xenograft overgrowth which was described in detail elsewhere [3,4]. Methylprednisolone was tapered down quickly and additional antihypertensive drugs (enalapril (Enahexal; Hexal, Holzkirchen, Germany) and metoprolol tartrate (Beloc; AstraZeneca, Cambridge, United Kingdom)) as well as the mTOR inhibitor temsirolimus (Torisel; Pfizer, New York, NY, USA) were administered.

### 2.5. Laboratory Tests

Blood samples were taken from baboon recipients prior to xenotransplantation, regularly during each experiment and before euthanasia. Effusion specimens were collected by thoracocentesis. Measurements were performed by the Institute of Laboratory Medicine (University Hospital, LMU Munich, Munich, Germany).

### 2.6. Assessment of Non-Gal-α(1,3)Gal Xenoreactive Antibody Levels

Plasma levels of baboon IgM and IgG directed against cellular antigens of Gal-α(1,3)-Gal-deficient pigs were measured by flow cytometry following the consensus protocol published previously [41]. In brief, *GGTA1-KO*, *hCD46/hTBM* transgenic porcine aortic endothelial cells (PAEC) were collected and suspended at 2 × 10^6^ cells per ml in staining buffer (PBS-1% BSA). Plasma samples were heat-inactivated at 56 °C for 30 min and diluted 1:20 in staining buffer. PAEC were incubated with diluted baboon plasma for 15 min at 37 °C. Cells were then washed with cold staining buffer and incubated with goat anti-human IgM-PE (SouthernBio, CAT: 2020-09; SouthernBiotech, Birmingham, AL, USA) or goat anti-human IgG-FITC (Invitrogen, Ref: 62-8411; Thermo Fisher Scientific, Waltham, MA, USA) for 1 h at 4 °C. After rewashing with cold staining buffer, cells were resuspended in PBS, fluorescence was acquired on FACS LSRII (BD Biosciences, Franklin Lakes, NJ, USA) and data were analyzed using FlowJo analysis software for detection of mean fluorescence intensity (MFI) in the FITC channel or in the PE channel. Data were then plotted using Prism 9 (GraphPad, San Diego, CA, USA).

### 2.7. Cytokine Secretion Profile

Systemic cytokine and chemokine levels (Eotaxin, IL-6, IL-12p70, I-TAC, MCP-1, MIG and TNF-α) were measured in sera and pleural effusions of the animals prior to xenotransplantation or at different timepoints after xenotransplantation. For cytokine and chemokine detection, an Invitrogen™ ProcartaPlex™ NHP Cytokine & Chemokine Panel, 29 plex assay (ThermoFisher Scientific) and a Luminex^®^ 100/200 analyzer (Luminex Corporation, Austin, TX, USA) were used. Standards and samples were prepared according to the manufacturer’s instructions and cytokine concentrations were calculated by Xponent software version 3.1 (Luminex Corporation).

### 2.8. Necropsy and Histology

Necropsies and histological analyses were performed at the Institute of Pathology (Faculty of Medicine, LMU Munich, Munich, Germany). Specimens were fixed in formalin, embedded in paraffin and plastic, sectioned and stained with hematoxylin and eosin using standard procedures. Grocott methenamine silver staining was performed for better visualization of fungal structures.

### 2.9. Immunohistochemical Staining

Myocardial tissue was fixed with 4% formalin overnight, paraffin-embedded and 3 µm sections were cut and dried. Heat-induced antigen retrieval was performed in Target Retrieval solution (DAKO S1699, Agilent Technologies, Santa Clara, CA, USA) in a boiling water bath for 20 min for detection of *hTBM* and in citrate buffer, pH 6.0, in a steamer for 45 min for detection of *hTBM*, respectively. Immunohistochemistry was performed using the following primary antibodies: mouse anti-human CD46 monoclonal antibody (HM2103, Hycult Biotech, Uden, The Netherlands) and mouse anti-human thrombomodulin monoclonal antibody (sc-13164, Santa Cruz Biotechnology, Dallas, TX, USA). The secondary antibody was a biotinylated AffiniPure goat anti-mouse IgG (115-065-146, Jackson ImmunoResearch, West Grove, PA, USA). Immunoreactivity was visualized using 3,3-diaminobenzidine tetrahydrochloride dihydrate (DAB; brown color). Nuclear counterstaining was performed with hemalum (blue color). In addition, routine immunohistochemical analysis for complement component C4d (C4d Polyclonal Antibody, Invitrogen/Thermo Fisher Scientific) was performed on all specimens.

### 2.10. Immunofluorescence Staining

Myocardial tissue samples were embedded in TissueTek O.C.T compound (Sakura 4583, Sakura Finetek, Torrance, CA, USA), and 10 μm thick sections were fixed and permeabilized with ice-cold 1:1 acetone (141007.1211, AppliChem, Darmstadt, Germany)/methanol (1.06009.2500, Merck Millipore, Darmstadt, Germany) for 10 min at room temperature and rehydrated in TBS for 5 min. After blocking for one hour at room temperature with TBS-3%BSA (Merck A7030, Merck Millipore), cryosections were incubated overnight at 4 °C with anti-porcine CD31 antibody (MAB33871, R&D Systems, Minneapolis, MN, USA), monoclonal anti-complement component C5b (InvitrogenDIA 011-01-02, clone aE11, Thermo Fisher Scientific), anti-human C3b/c (DAKO A0062, Agilent Technologies), anti-human C4b/c (DAKO F0169, Agilent Technologies), anti-human IgM (Sigma F 5384, Merck Millipore), polyclonal anti-human fibrinogen (DAKO F0111, Agilent Technologies), wheat germ agglutinin (WGA from triticum vulgaris L4895 Sigma, Merck Millipore), anti-human Von Willebrand Factor (VWF DAKO A0082, Agilent Technologies), and anti-human CD68 (ab955, Abcam, Cambridge, United Kingdom) diluted in TBS-1%BSA-0.05% Tween (Tween 20, A4974,0250, AppliChem). Subsequently, samples were incubated for 1 h under agitation at room temperature with secondary antibodies: goat anti-rat IgG AlexaFluor 680 (Invitrogen A21096, Thermo Fisher Scientific), goat anti-rat IgG AlexaFluor 568 (Invitrogen A11077, Thermo Fisher Scientific), goat anti-rabbit IgG AlexaFluor 568 (Invitrogen A11036, Thermo Fisher Scientific), donkey anti-mouse AF 568 (Invitrogen A10037, Thermo Fisher Scientific), and goat anti-mouse IgG AlexaFluor 488 (Invitrogen A32766, Thermo Fisher Scientific). All secondary antibodies were diluted in TBS-1%BSA-0.05%Tween. Slides were washed, mounted with Prolong Diamond Antifade Mountant with DAPI (Invitrogen P36962, Thermo Fisher Scientific) and imaged using a 20x objective on a Zeiss LSM980 confocal microscope (Zeiss, Oberkochen, Germany) and analyzed with Image J (version 2.3.0/1.53q).

### 2.11. Statistics

Data collection and analyses were performed with Excel (Microsoft, Redmond, WA, USA) and Prism 9.0 (GraphPad). For survival data, Kaplan–Meier curves were plotted and the Mantel–Cox log-rank test was used to determine significant differences between groups. Data are presented either as single measurements or as group means ± standard deviations (s.d.).

## 3. Results

Here, we present the results from orthotopic cardiac pig-to-baboon xenotransplantation experiments with an immunosuppressive therapy based on a combination of CD40 and CD40L blockade. We compare this combination therapy to a previously published study group treated only with the anti-CD40 Mab as co-stimulation blockade [4]. Hearts from six genetically modified piglets, homozygous for α1,3-galactosyltransferase knockout (*GGTA1-KO*) and hemizygous transgenic for human CD46 (*hCD46*) as well as human thrombomodulin (*hTBM*), were transplanted into male captive-bred baboons. In addition to an initial B cell depletion therapy with rituximab, all animals were continuously dosed with a combination of an anti-CD40 IgG4 (initially 50 mg/kg body weight (bw), lowered to 30 mg/kg bw) and the anti-CD40L PAS-Fab (initially 20 mg/kg bw, lowered to 10 mg/kg bw) (Figure 1).

### 3.1. Recipient Survival Times

Mean survival in the current combination therapy group was 78 days, and maximum survival was 170 days (Table 1). Hence, survival rates in the six xenotransplantation experiments were not significantly different from the results of a previous group treated with an immunosuppressive regimen based on monotherapy with the same anti-CD40 Mab alone [4] (*p* = 0.0896, Log-rank (Mantel–Cox) test, *n* = 10; Figure 1).

Two of the six experiments (#16950 and #17353) had failed early due to technical reasons. These two animals were excluded from further data analysis. Two more experiments (#16956 and #16935) were deliberately terminated when the predetermined period of 90 postoperative days (set by the regulatory authorities) was reached with both recipients in excellent clinical condition. The final two experiments (#17012 and #17020) were extended beyond 90 days (with permission from the regulatory authorities). Animal #17012 developed pleural effusions and was euthanized after 120 days. Animal #17020 appeared in good clinical condition until day 165. Thereafter, the cardiac function deteriorated rapidly due to acute humoral rejection, and the animal was euthanized after 170 days.

### 3.2. Organ Blood Parameters and Non-Gal-α(1,3)-Gal Xenoreactive Antibody Levels

**Figure 2:**
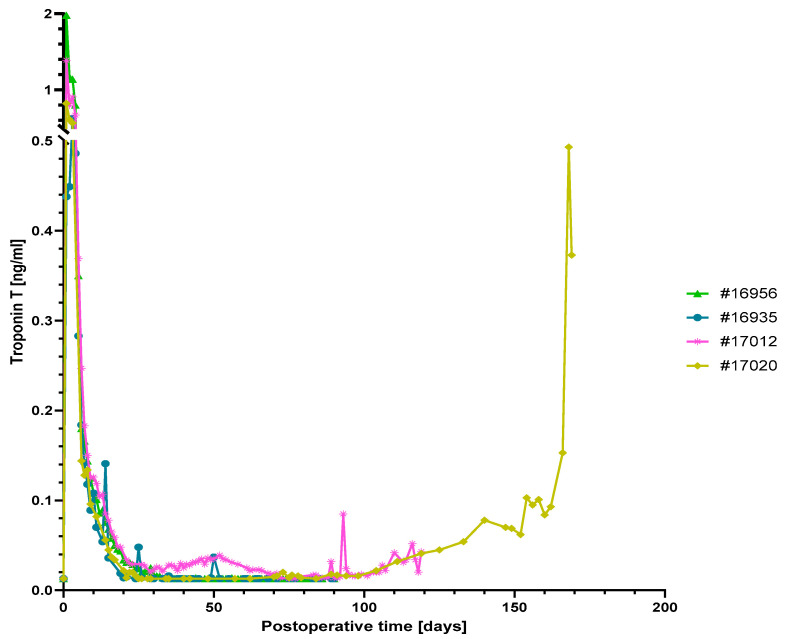
Serum troponin T levels. Levels were high after surgery but subsequently dropped to a normal range in animals #16956, #16935 and #17012. The strong increase in serum troponin T levels in animal #17020 indicates myocardial damage due to graft rejection towards the end of the experiment.

Serum levels of the cardiac enzyme troponin T were high after surgery but dropped subsequently to a normal range within the first postoperative week in all animals (Figure 2). In the two experiments which were electively terminated after 90 days (#16956 and #16935), troponin T levels were within a normal range throughout the study.

In the two animals that were extended beyond 90 days, troponin T levels gradually increased towards the end of the experiment (Figure 2); this increase was especially pronounced in baboon #17020 after day 160, thus indicating myocyte damage due to acute humoral rejection. In baboon #17012, an early moderate increase in troponin T levels coincided with the appearance of pleural effusion on day 30 (see below); however, at day 70 troponin T levels had returned to the normal range.

**Figure 3:**
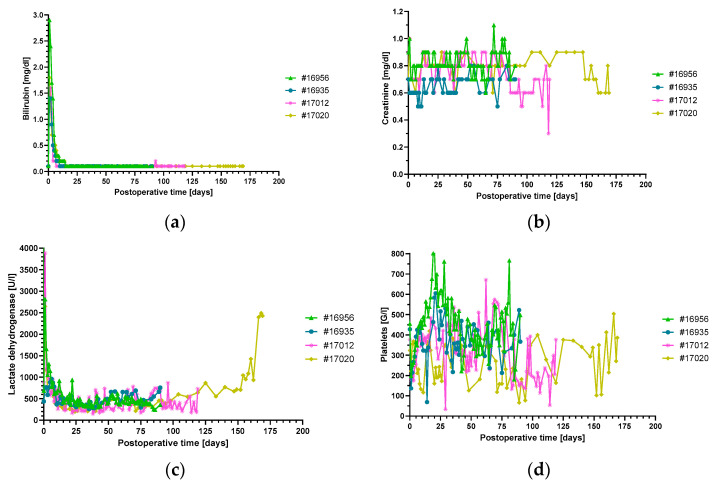
Plasma bilirubin (**a**), creatinine (**b**) and LDH (**c**) levels and platelet counts (**d**), non-suggestive of thrombotic microangiopathy. There were no signs of liver or kidney damage. The increase in LDH in animal #17020 at the end of the experiment was caused by humoral rejection. LDH, lactate dehydrogenase.

Other laboratory markers such as platelets, LDH, creatinine and bilirubin showed a mostly inconspicuous time course in all experiments (Figure 3).

**Figure 4:**
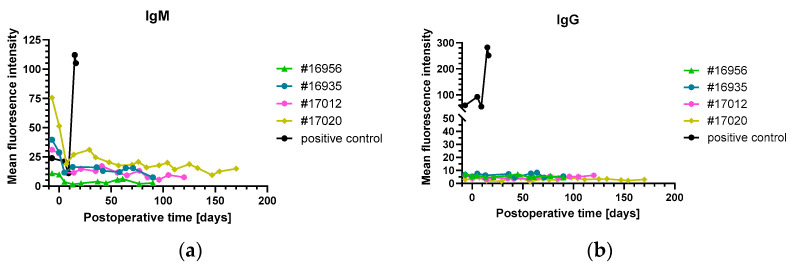
Levels of non-Gal-α(1,3)-Gal xenoreactive IgM (**a**) and IgG (**b**) as measured by flow cytometry on porcine aortic endothelial cells (PAEC) from *GGTA1-KO*, *hCD46/hTBM* transgenic animals. The values from a previously investigated baboon that had rejected an intrathoracic heterotopically transplanted pig heart served as positive control (black) and showed a strong increase in both IgM and IgG, indicative of humoral rejection. Note: while animal #17020 revealed clinical and histological signs of humoral rejection, there was no increase in xenoreactive IgM and IgG.

Only in baboon #17020, LDH levels started to increase after 100 days with a sharp peak toward the end of the experiment, coinciding with the marked increase in troponin T level (Figure 2 and Figure 3c). Notably, the analysis of IgM or IgG directed against non-Gal-α(1,3)-Gal PAEC from *GGTA1-KO*, *hCD46/hTBM* transgenic animals showed no relevant increase in all experiments (Figure 4).

### 3.3. Levels of Inflammatory and Injury Markers

**Figure 5:**
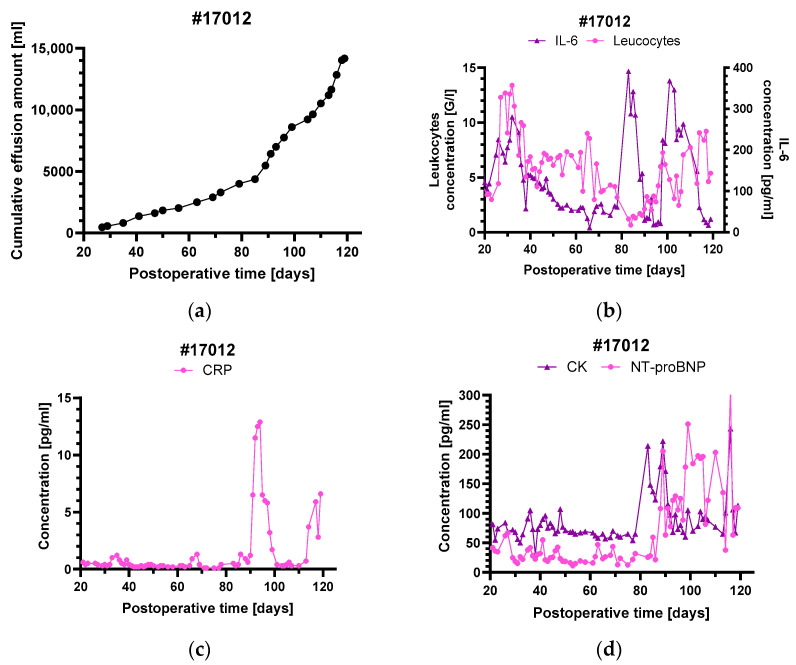
Cumulative pleural effusions in baboon #17012 (**a**) and serum levels of the inflammatory markers leukocytes and IL-6 (**b**), CRP (**c**) as well as the myocardial markers CK and NT-proBNP (**d**). The sharp rise in pleural effusions around postoperative day 85 was accompanied by a marked increase in all inflammatory and myocardial markers ((**a**–**d**) and Figure 2). For comparison, at the beginning of the pleural effusions around postoperative day 30 there was only an increase in IL-6, leukocyte count (**b**) and also in troponin T levels (Figure 2).

In contrast to the other animals, baboon #17012 developed recalcitrant pleural effusions during the experiment (Figure 5a). Beginning around postoperative day 30, effusions were initially mild and were controlled by occasional drainage. This first phase was accompanied by a sharp increase in leukocytes and serum IL-6 levels but no relevant increase in CRP (Figure 5b,c). The occurrence of pleural effusions also coincided with the first rise in troponin T level (Figure 2), while other myocardial markers like NT-proBNP or CK remained stable (Figure 5d).

**Figure 6:**
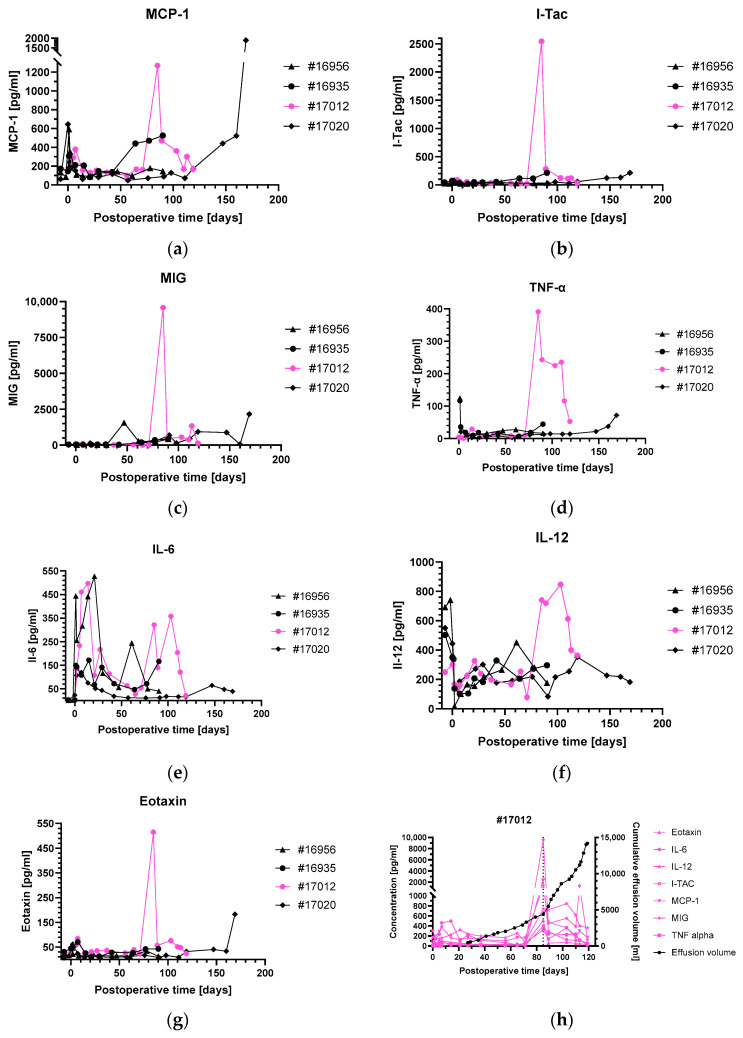
Serum levels of different pro-inflammatory cytokines in all animals of the study group (**a**–**g**). The marked increase around postoperative day 85 was only observed in baboon #17012 (magenta), whereas #17020 showed elevated levels of some cytokines towards the end of the experiment, when organ rejection occurred. The sharp increase in pleural effusions around postoperative day 85 in baboon #17012 (**h**) was accompanied by a strong rise in several pro-inflammatory cytokine levels (magenta).

After day 85, there was a sudden sharp rise in pleural effusions, needing more regular thoracocentesis. This second phase was also accompanied by increases in leukocyte numbers, IL-6 (Figure 5b) and troponin T (Figure 2). In contrast to the first phase of pleural effusions around day 30, there were also relevant increases in NT-proBNP, CK, CRP (Figure 5c,d) as well as several chemokines and pro-inflammatory cytokines, like MCP-1, I-TAC, MIG, IL-6, IL-12, TNF-α and eotaxin (Figure 6). The other animals did not show any relevant changes in chemokines and pro-inflammatory cytokines over the entire course of the experiment, except for MCP-1 in baboons #16935, #17012 and, starting after day 110, MCP-1, MIG, TNF-α and eotaxin in baboon #17020 (Figure 6a–g).

### 3.4. Necropsy and Histology

**Figure 7:**
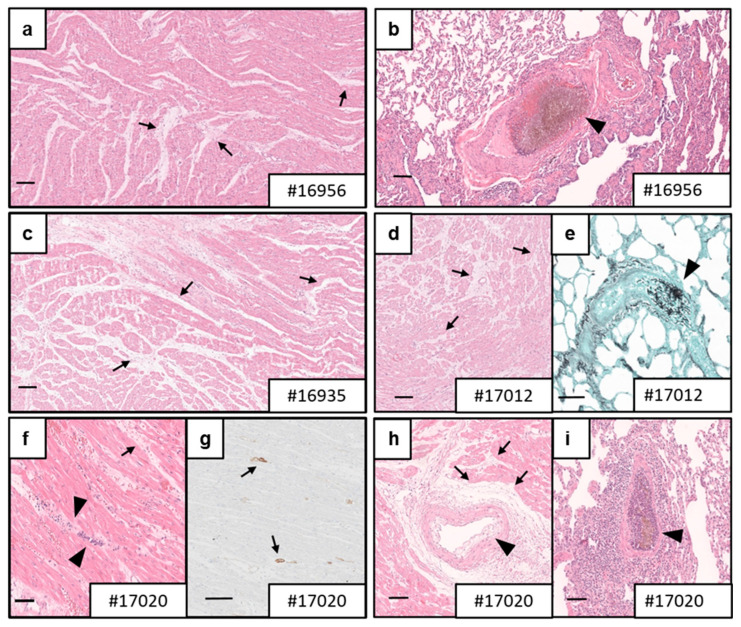
Microscopic findings in post-mortem myocardial specimens. Histological analysis revealed mild-to-marked interstitial edema (arrows) in myocardial specimens of baboons #16956 (**a**), #16935 (**c**), #17012 (**d**) and #17020 (**f**,**h**). Fungal thrombi in pulmonal vessels (arrowhead) were detected in all animals (#16956, (**b**); #17012, (**e**); #17020, (**i**); not shown for #16935). Based on capillaritis (arrowhead, (**f**)), endothelial swelling (arrowhead, (**h**)) and elongated C4d staining in capillaries (arrows, (**g**)), baboon #17020 was diagnosed with severe antibody-mediated rejection (AMR3). (**a**–**d**,**f**,**h**,**i**), H&E staining; (**e**), Grocott methenamine staining; (**g**), C4d staining; scale bars = 100 µm.

Histopathological analysis of the transplanted transgenic porcine hearts revealed no evidence of antibody-mediated or cellular rejection in three of the four animals that were under continued study (Figure 7a–e). However, the transplanted heart in baboon #17020 showed focal capillaritis, interstitial edema, hemorrhages, acute myocardial necrosis and distinct C4d positivity of capillaries, which are histological signs of severe antibody-mediated rejection (pAMR3 according to the classification by the International Society for Heart and Lung Transplantation, ISHLT) (Figure 7f–i).

Notably, all animals showed signs of fungal colonization in the lungs (Figure 7b,e,i; not shown for baboon #16935). In addition, fungal emboli caused by yeast cells and hyphae were sporadically found in lung arteries of baboon #16956; however, deep mycosis was not detected (Figure 7b). While micromorphology did not allow for a specific diagnosis, size and morphology were consistent with *Candida* species. The livers of baboons #16956, #16935 and #17020 showed hepatic steatosis to varying degrees. The diagnostic workup of the other organs (e.g., kidneys, spleen, intestine) did not reveal any clinically relevant findings.

### 3.5. Analyses of Complement System, Coagulation System, Tissue Structure and Innate Immune Cell Infiltration

**Figure 8:**
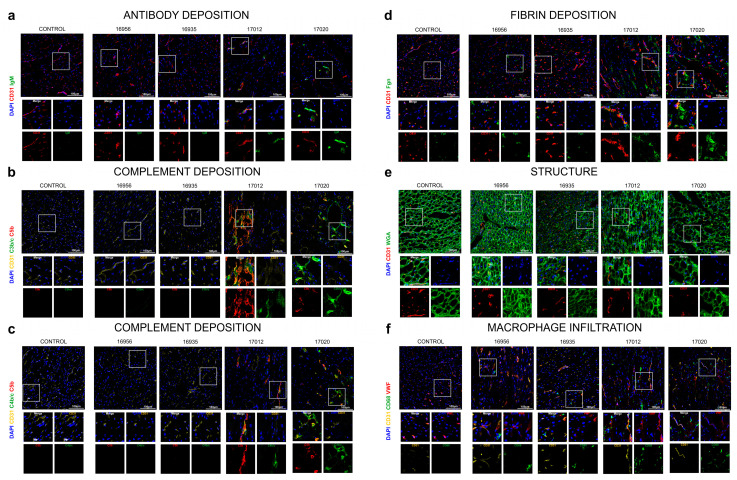
Immunofluorescence staining of post-mortem myocardial specimens. Antibody deposition (IgM) (**a**), complement deposition (C3b/c, C4c, C5b) (**b**,**c**), fibrin deposition (Fgn) (**d**), cardiomyocyte structure (WGA, a lectin staining N-acetyl-D-glucosamine and sialic acid on the cell membrane) (**e**) and macrophage infiltration (CD68) (**f**) were analyzed in all donor organs. Scale bar, 100 µm. *n* = 4 *GGTA1-KO*, *hCD46/hTBM* transgenic pigs; *n* = 1 wild-type pig (control).

In the two experiments deliberately terminated after 90 postoperative days (#16956 and #16935), immunofluorescence staining did not reveal notable antibody deposition, complement activation, fibrin deposition or cellular infiltrates (macrophages) compared to a non-transplanted, wild-type pig heart as the control (Figure 8a–d,f). The myocardial tissues were overall healthy, with interspersed capillary distribution, well-organized polygonal cardiomyocytes and discernible rounded nuclei (Figure 8e).

In the two experiments extended beyond 90 postoperative days (#17012 and #17020), the immunofluorescence results showed a different pattern. A mild IgM antibody deposition and activation of the complement system (C4b/c, C3b/c and C5b, Figure 8a–c) were clearly visible. Mild vascular and extravascular fibrin depots correlated with increased structural alterations of the myocardial tissue as assessed by membrane staining with WGA lectin (Figure 8d,e). Interestingly, there was also a massive infiltration of macrophages (Figure 8f).

### 3.6. Virus Testing

The donor pigs as well as the baboon recipients were tested for PCMV/PRV using real-time PCR, nested-PCR and PCMV/PRV glycoprotein B (gB)-based peptide ELISA and Western blot assays. All tested organs of the baboons (skin, kidney, spleen, liver, lung, peripheral blood mononuclear cells) as well as the explanted transgenic pig heart were negative for PCMV/PRV as well as PCV3 and PLHV-3. Preventing the transmission of PCMV/PRV, PLHV-3 and PCV3 to the baboon recipients significantly contributed to their survival because pig hearts infected with PCMV/PRV typically survive less than 30 days [42]. Since the antiviral substances ganciclovir and valganciclovir are effective against human cytomegalovirus (HCMV) but not against PCMV/PRV, which is a roseolovirus [43,44,45], they should not be administered for the prevention of a PCMV/PRV infection. Of note, PCMV/PRV was also transmitted to the first recipient of a pig heart in Baltimore [8,9] and may have contributed to the early death of this patient.

## 4. Discussion

There are several studies demonstrating the therapeutic potential of agents interfering with the CD40/CD40L immune checkpoint, but it is still unclear whether a single Mab or a combination of two or more Mabs may be better at preventing xenograft rejection [10].

### 4.1. Rationale for a Combination Therapy with Anti-CD40 and Anti-CD40L Antibodies

When using an anti-CD40 Mab for co-stimulation blockade in preclinical non-human primate cardiac xenotransplantation models, a dosage of 50 mg/kg bw seemed appropriate for long-term xenograft survival [14,15,21]. When lower dosages were given [21], or when the dosage was tapered out over the course of the experiment [14,15], xenografts were ultimately rejected. However, the high dosage of 50 mg/kg bw might not be feasible in future human applications (amounting to several grams of antibody to be administered per dose). As described above, a combination of the anti-CD40 and anti-CD40L antibodies may have synergistic effects in vivo, which should allow dose reduction for each agent. To date, a combination of anti-CD40 and anti-CD40L Mabs has not been published, and we present the first description of such an approach.

Thrombotic side effects were described for the first-generation anti-CD40L Mab [16,17] and, consequently, the clinical development of ruplizumab (hu5c8) was halted. As the Fc-part of the Mab was found to be the cause of this detrimental effect [17,46], this portion had been removed in the anti-CD40L PAS-Fab which was used in the current study. The resulting shorter plasma half-life of the monovalent antibody fragment—which also offers the functional benefit that it cannot cross-link its antigen—was extended using PASylation technology [33]. This novel immunosuppressive agent was successfully applied as single therapy in xenotransplantation studies before [3].

### 4.2. Outcome of a Combination Therapy of Anti-CD40 IgG4 and Anti-CD40L PAS-Fab

We initially administered both antibody agents at high dosages, which were decreased after 30 or 60 days, respectively. The aim of this procedure was to reduce the amount of drug substance administered to the patient in a potential clinical setting. The results of this animal study proved that, in principle, a combination therapy of anti-CD40 and anti-CD40L is effective and well tolerated (except for the fungal infections, see below). There was no significant difference in survival between the current combination therapy group and a previous study group using only the anti-CD40 Mab [4]. Three of four animals showed rejection-free survival for up to 120 days. No large-vessel thrombogenic complications were seen in any animal of the study group. This needs to be emphasized, as thrombotic side effects were described for a first-generation anti-CD40L Mab [16,17]. Nevertheless, the fourth animal succumbed to acute humoral rejection after 170 postoperative days.

During the first 90 postoperative days, there was no relevant difference in clinical appearance, vital signs or laboratory analyses among the four animals investigated here and compared to previous study groups using either the anti-CD40 IgG4 or anti-CD40L PAS-Fab [3,4]. Only the two present experiments extending beyond 90 days (#17012 and #17020), i.e., 60/30 days after decreasing antibody doses, exhibited a conspicuous course, as demonstrated, for example, by increasing serum levels of LDH, AST (#17020) and troponin T (#17012 and #17020, see below). This is consistent with previous data, when serum LDH of >600 U/L and AST of >300 U/L were associated with the development of acute humoral rejection after cardiac xenotransplantation [47].

Interestingly, animal #17020, which suffered from severe humoral rejection, showed no relevant increase in non-Gal-α(1,3)-Gal xenoreactive IgM or IgG in the plasma. Of note, the immunochemical assay applied [41] only served to detect non-donor-specific circulating antibodies directed against surface antigens of PAEC from the *GGTA1-KO*, *hCD46/hTBM* transgenic donor pigs, but not those reactive with intracellular or secreted porcine proteins. This constellation was also observed in heterotopic abdominal cardiac xenotransplantation experiments when non-Gal-α(1,3)-Gal xenoreactive antibodies were not always detected at the time of graft or recipient demise [14]. Nevertheless, the authors of that study hypothesized that there was incomplete control of anti-pig immunity in these animals [14].

In contrast, immunofluorescence analyses of the myocardial tissue from animal #17020 revealed the deposition of IgM antibodies and of complement (C4b/c, C3b/c, and C5b). There are two possible explanations for these apparently contradictory findings: (i) The anti-non-Gal-α(1,3)-Gal xenoreactive antibodies produced by the baboon may almost entirely be absorbed by the porcine cardiac xenograft and are therefore difficult to be detected in plasma. (ii) The antibodies could be specific for the SLA-II antigen of the donor and would not be detected by FACS with *GGTA1-KO*, *hCD46/hTBM* transgenic PAEC, as SLA-II is only expressed on activated porcine endothelium. Further experiments are therefore needed to identify the targets of the putative anti-donor antibodies, and the currently used assay needs to be adapted or complemented with specific detection of anti-SLA-II.

Another interesting finding was a correlation between the courses of increased MCP-1 levels and macrophage infiltration in three of the four animals, i.e., #16935, #17012 and #17020. The involvement of macrophages is a prominent feature of the innate immune response and has been described in xenograft rejection before [48]. MCP-1 (monocyte chemoattractant protein-1) is one of the key chemokines that regulate migration and infiltration of monocytes/macrophages into inflammatory tissue sites and, thus, elevated levels of MCP-1 may play a crucial role in graft survival [49]. While prevention of graft rejection mediated by innate immune cells was not in the focus of the present study, this needs to be investigated more closely in future experiments.

### 4.3. Myocardial Integrity of the Xenografts

As mentioned above, a conspicuous course of the myocardial integrity marker troponin T was only present in the two experiments extended beyond 90 days, #17012 and #17020. The increase in troponin T after day 100, i.e., 70/40 days after decreasing the antibody doses, was nearly identical in both animals and correlated with tissue damage detectable as mild vascular and extravascular fibrin depots with increased alteration of the myocardial structure. We assume that these changes should be considered in the context of a multifactorial genesis.

In part, the increase in troponin T levels after day 100 could have been caused by an insufficient immunosuppression as a result of reducing the doses of both anti-CD40 Mab and anti-CD40L PAS-Fab. In a published heterotopic abdominal model, the reduction in the anti-CD40 Mab monotherapy dose resulted in the recrudescence of anti-pig antibody and graft failure [14]. In our current study group, we cannot clarify whether decreasing the dose of either the anti-CD40 Mab, the anti-CD40L PAS-Fab, or of both agents, may have caused the pathological observations. However, the assumption of an insufficient immunosuppression is supported by the fact that necropsy of baboon #17020 showed signs of severe humoral rejection.

The changes in troponin T levels could also have been caused by disproportionate myocardial growth or xenogeneic hypertrophic obstructive cardiomyopathy [50]. This phenomenon was strongly attenuated, but not totally stopped, by our regimen of growth inhibition, including temsirolimus, antihypertensive treatment and early weaning from cortisone [3,50]. Furthermore, fungal infections (see below) are also known to cause elevation of troponin T levels [51,52].

### 4.4. Pleural Effusions in Animal #17012

The development of pleural effusions in animal #17012 was seen in two phases, which both came along with an increase in leukocyte numbers, IL-6 and troponin T levels. During the second phase, there was an additional increase in NT-proBNP, CK, CRP as well as several pro-inflammatory cytokines. The rise in leukocytes, CRP and IL-6 indicates the occurrence of inflammatory processes coinciding with the development of the pleural effusions. Furthermore, the massive complement deposition observed in baboon #17012 leads us to the assumption that complement activation may also have contributed to the pleural effusions. Besides this, some myocardial damage seems to have occurred, which is indicated by the concomitant increase in troponin T, NT-proBNP and CK.

To our knowledge, there is no long-term data on pro-inflammatory cytokines after orthotopic cardiac xenotransplantation available so far. In cardiac allotransplantation recipients, levels of chemokines and pro-inflammatory cytokines increase in the months following transplantation, possibly mediating endothelial damaging [53] and the recruitment of more xenoreactive immune cells into the graft [54]. Chemokines such as I-TAC or MIG are modulators of intragraft inflammation by recruiting CXCR3^+^ T-cells [55,56]. Furthermore, it is known that pro-inflammatory cytokines such as TNFα may affect endothelial function [57,58]. In lung allotransplantation, pro-inflammatory cytokines seem to be involved in transplant outcome [59,60] and are associated with pro-inflammatory responses leading to primary graft dysfunction [61]. Moreover, previous studies in murine models of allogeneic cardiac transplantation indicated that antibodies blocking cytokines such as IL-12 support graft survival by preventing Th1 and Th17 responses [62].

Taking these findings together, there might have been several factors responsible for the recalcitrant effusions in baboon #17012: infectious/inflammatory processes, myocardial damage, endothelial dysfunction and insufficient immunosuppression. Our analysis is the first of its kind and therefore provides important clues regarding pleural effusions as a challenge after pig-to-baboon cardiac xenotransplantation. Further studies, especially with a larger number of animals, should help to gain a better understanding of the incidences, causes and consequences of pleural effusions after pig-to-baboon cardiac xenotransplantation.

### 4.5. Fungal Infections

Necropsy data of all four animals of the current study group showed fungal infections, which was unexpected, especially as the animals did not show any corresponding clinical signs throughout the experiments. Fungal infections of such significant extent were not observed in other xenografted animal groups studied in our laboratory (data partially not published) [3,4,63,64] or in experiments reported by other research groups [5,14,65]. Therefore, antifungal prophylaxis was not part of the therapeutic regimen in the current study group. For comparison, in cardiac, lung, liver or pancreas allotransplantation, patients have a relevant incidence of 1% to 40% of fungal infections [66,67,68]. In clinical practice, this can be anticipated by antifungal prophylaxis, which is well established in human allotransplantation [68,69,70] and may also be an option for future preclinical and clinical xenotransplantation.

Beyond the anti-CD40 IgG4/anti-CD40L PAS-Fab combination therapy, all animals received mycophenolate mofetil as a generally immunosuppressive drug, which is known to increase the risk of fungal infections [71,72,73]. However, since the animals in the aforementioned studies [3,4,5,14,63,64,65] had also received this drug, we assume that the fungal infections were more likely caused by the antibody combination therapy. CD40/CD40L signaling is known to contribute to the adaptive Th1 immune response against fungi, such as *Candida albicans*, and life-threatening fungal infections were reported in patients deficient in CD40L or CD40 [74,75]. Indeed, the higher susceptibility for infectious side effects by all treated animals may provide an indication that the suppression of this particular immune checkpoint was overly strong in our study.

Interestingly, fungal infections were only diagnosed through necropsy and histological examination after the experiments had been stopped. During the experiments, there were no specific signs of fungal infections, such as in blood culture examinations or other samples. Consequently, determining the precise onset of these infections is challenging. We assume that they likely originated during the period prior to reducing the doses of the combination therapy, resulting in an overly strong cellular and humoral immunosuppressive effect. The pathologies in animals #17012 and #17020 (i.e., pleural effusions, increase in inflammatory markers, humoral rejection, see above) clearly manifested after decreasing the anti-CD40 IgG4 and anti-CD40L PAS-Fab doses, and there were no comparable events in the animals #16956 and #16935, which were deliberately terminated after 90 postoperative days. It is tempting to speculate that the ’ideal’ dosage for the combination therapy of anti-CD40 IgG4 and anti-CD40L PAS-Fab may fall within the range between the high (promoting fungal infections in all animals) and low dosages (permitting humoral rejection and recalcitrant pleural effusions) administered in the current study group. This aspect should be addressed in future studies on a combination therapy of antibodies blocking both CD40 and CD40L. Besides fungal infections, xenotransplantation with corresponding anti-CD40-based immunosuppression can be associated with the activation of latent herpes viruses, including HCMV, as well as external infections such as *Pneumocystis* pneumonia [76,77]. However, it is quite feasible to diagnose and treat these infections.

## 5. Conclusions

Our experience with the first combination therapy of anti-CD40 and anti-CD40L antibodies proved that such therapy is effective and generally well-tolerated. However, there were also side effects/adverse events like humoral rejection, susceptibility to fungal infections and recalcitrant effusions. Given the critical role of dosage in the balance between infection and organ rejection, further trials and broader immunologic monitoring are necessary to obtain a deeper understanding of the potential benefits of an anti-CD40 and anti-CD40L co-stimulation blockade in preclinical cardiac xenotransplantation.

## Figures and Tables

**Table 1:** Serum levels of heart and liver enzymes, platelet counts and prothrombin ratio at the end of all six experiments.

Experiment	#16956	#16935	#16950	#17353	#17012	#17020	Reference *
**Bilirubin [mg/dL]**	<0.1	<0.1	5.8	5.8	<0.1	<0.1	≤1.2
**AST [U/L]**	27	64	1360	332	56	456	≤49
**PR [%]**	88	128	13	41	141	60	70–130
**CHE [kU/L]**	112	13.4	1.2	1.9	1.6	8.6	4.6–11.5
**Trop. T [ng/mL]**	<0.013	<0.013	10.30	4.85	0.043	0.373	≤0.014
**CK total [U/L]**	47	79	18689	5826	116	501	≤189
**LDH [U/L]**	361	757	13630	1028	729	2429	≤249
**Platelets [G/L]**	498	498	367	42	118	386	150–300
**Survival [days]**	90	90	1	1	120	170	
**Causes for euthanasia**	Study endpoint	Study endpoint	Technical failure (pulmonary stenosis)	Technical failure (insufficient perfusion)	Recalcitrant pleural effusions	Graft failure (humoral rejection)	

AST, aspartate aminotransferase; CHE, cholinesterase; Trop. T, troponin T; CK, creatine kinase; LDH, lactate dehydrogenase; PR, prothrombin ratio. The two experiments #16950 and #17353 were excluded from further data analysis as they had to be stopped within the first 24 h due to technical failures. *, Reference values as defined by the Institute of Laboratory Medicine (University Hospital, LMU Munich, Munich, Germany).

## Data Availability

The raw data supporting the conclusions of this article will be made available by the authors on request.
